# Advances in *Physalis* molecular research: applications in authentication, genetic diversity, phylogenetics, functional genes, and omics

**DOI:** 10.3389/fpls.2024.1407625

**Published:** 2024-06-27

**Authors:** Yan Jiang, Yanyun Jin, Yiyi Shan, Quanzhou Zhong, Huizhong Wang, Chenjia Shen, Shangguo Feng

**Affiliations:** ^1^ Hangzhou Normal University, Hangzhou, China; ^2^ Zhejiang Provincial Key Laboratory for Genetic Improvement and Quality Control of Medicinal Plants, Hangzhou Normal University, Hangzhou, China

**Keywords:** genetic diversity, molecular marker, inflated calyx syndrome, omics, *Physalis*

## Abstract

The plants of the genus *Physalis* L. have been extensively utilized in traditional and indigenous Chinese medicinal practices for treating a variety of ailments, including dermatitis, malaria, asthma, hepatitis, and liver disorders. The present review aims to achieve a comprehensive and up-to-date investigation of the genus *Physalis*, a new model crop, to understand plant diversity and fruit development. Several chloroplast DNA-, nuclear ribosomal DNA-, and genomic DNA-based markers, such as *psbA-trnH*, internal-transcribed spacer (ITS), simple sequence repeat (SSR), random amplified microsatellites (RAMS), sequence-characterized amplified region (SCAR), and single nucleotide polymorphism (SNP), were developed for molecular identification, genetic diversity, and phylogenetic studies of *Physalis* species. A large number of functional genes involved in inflated calyx syndrome development (*AP2-L*, *MPF2*, *MPF3*, and *MAGO*), organ growth (*AG1*, *AG2*, *POS1*, and *CNR1*), and active ingredient metabolism (*24ISO*, *DHCRT*, *P450-CPL*, *SR*, *DUF538*, *TAS14*, and *3β-HSB*) were identified contributing to the breeding of novel *Physalis* varieties. Various omic studies revealed and functionally identified a series of reproductive organ development-related factors, environmental stress-responsive genes, and active component biosynthesis-related enzymes. The chromosome-level genomes of *Physalis floridana* Rydb., *Physalis grisea* (Waterf.) M. Martínez, and *Physalis pruinosa* L. have been recently published providing a valuable resource for genome editing in *Physalis* crops. Our review summarizes the recent progress in genetic diversity, molecular identification, phylogenetics, functional genes, and the application of omics in the genus *Physalis* and accelerates efficient utilization of this traditional herb.

## Introduction

*Physalis* L. is one of the largest genera within the Solanaceae family consisting of approximately 75–90 species, which are mostly distributed in tropical and temperate regions worldwide (Whitson and Manos, 2005; Pretz and Deanna, 2020). The most notable characteristic of the species in this genus is the calyx, which surrounds the fruit and increases in size as the fruit grows larger. The interest in *Physalis* species is mostly motivated by the economic importance of a subset of species that have been used in traditional medicine (Zhang and Tong, 2016). Most *Physalis* species have potential medicinal properties, including antibacterial, antileukemic, antipyretic, anti-inflammatory, immunomodulatory, and anticancer actions, and often have been used to treat various illnesses such as dermatitis, malaria, asthma, hepatitis, and liver disorders. Moreover, some *Physalis* species are extensively cultivated for their edible fruit or ornamental value in various countries. Recently, there has been a growing focus on the genus *Physalis* in molecular research related to taxonomy, systematics and evolution, genetic diversity, and omics. In this review, we aim to provide a comprehensive analysis of the genetic diversity, molecular identification, phylogenetics, functional genes, and the application of omics in the genus *Physalis*. The relevant references for this review were obtained from the PubMed database of NCBI and the Web of Science, which are widely recognized as leading databases for published articles and citations. The searches were conducted within a single day in May 2024. The term “*Physalis*” was utilized to search for instances in the title, abstract, and keywords. Articles focusing on authentication, genetic diversity, phylogenetics, functional genes, and omics were chosen for inclusion.

## Molecular authentication

The accurate identification of germplasm resources is a crucial foundation for the systematic classification, population genetics, omics research, and molecular genetic breeding of *Physalis* plants. In the past, morphological methods were the primary means of identifying *Physalis* plants (Sinha, 1951; Axelius, 1996; Gonzalez et al., 2008). However, the morphological characteristics of *Physalis* plants are very similar, and these morphological traits are extremely susceptible to the restrictions of growth period and growth environment, which bring great difficulties to morphological identification methods (Whitson and Manos, 2005; Feng et al., 2016). With the development of biotechnology, various molecular markers have emerged and been widely used in the identification of plant species, varieties, and genotypes. Compared with morphological methods, DNA molecular markers are not easily affected by the external environment and growth period, and their identification has good stability and high accuracy (Schindel and Miller, 2005; Sarwat et al., 2012; Li et al., 2015).

Several chloroplast DNA regions (such as *rbcL*, *atpF-atpH*, *ycf1*, *matK*, *rpoB*, and *psbA-trnH*) and some nuclear ribosomal DNA (nrDNA) regions [such as internal transcribed spacer, ITS, and internal transcribed spacer 2 (ITS2)] have been advocated by some experts and the Consortium for the Barcode of Life (CBOL) as potential standard DNA barcodes for plant species identification (Schindel and Miller, 2005; Cbol Plant Working Group, 2009; Chen et al., 2010; China Plant et al., 2011; Chen et al., 2024). Feng et al. (2016) demonstrated the efficacy of nrDNA ITS2 regions for molecular identification in 45 *Physalis* species, as detailed in **Table 1**. The findings revealed a high rate of species authentication using the ITS2 sequence suggesting its potential as an efficient barcode for identifying *Physalis* species. Furthermore, the variability in secondary structures of ITS2 among most *Physalis* species, including differences in loop number, size, position, and degree of angles from the center of the spiral arm, presents a novel approach for identifying challenging-to-distinguish species based on their ITS2 sequence. Terrones et al. (2021) successfully used nrDNA ITS sequences as a DNA barcode to authenticate the two species of the genus *Physalis* [*Physalis acutifolia* (Miers) Sandwith and *Physalis angulata* L.] in the Iberian Peninsula of Spain (Terrones et al., 2021). Chloroplast *psbA-trnH* region, as one of the highly recommended candidate DNA barcodes, had also been successfully applied to the molecular identification of the species of the genus *Physalis* (Feng et al., 2018a).

**Table 1:** Molecular techniques applied to *Physalis* authentication, genetic diversity, and phylogenetics.

Physalis species	Molecular marker	Study type	Publication	Group/year
45 *Physalis* species	nrDNA ITS2	Molecular authentication and phylogenetics	*Front Plant Sci*	Feng et al., 2016
*P. acutifolia* (Miers) Sandwith/*P. angulata* L.	ITS	Molecular authentication	*Annali Di Botanica*	Terrones et al., 2021
Eight *Physalis* species	*psbA-trnH*	Molecular authentication	*Genome*	Feng et al., 2018a
*P. peruviana* L./*P. floridana* L.	SSR	Molecular authentication	*PLoS One*	Simbaqueba et al., 2011
*P. ixocarpa* Brot. ex Hornem.*/P. peruviana* L.	RAMS	Molecular authentication	*Revista De Ciencias Agricolas*	Delgado-Alvarado et al., 2018
Four *Physalis* species	SCoT, SCAR	Molecular authentication	*Front Genet*	Feng et al., 2018b
*P. angulata* L.	RAPD	Genetic diversity	*Pertanika Journal of Science and Technology*	Hidayat et al., 2017
*P. ixopcarpa* L.	RAPD	Genetic diversity	*PLoS One*	Khan et al., 2019
*P. peruviana* L.	SSR	Genetic diversity	*Revista De Ciencias Agricolas*	Delgado-Bastidas et al., 2019
*P. angulata* L.	SSR	Genetic diversity	*Plants*	Feng et al., 2023
Eight *Physalis* species	ISSR	Genetic diversity	*Nutrients*	Vargas-Ponce et al., 2011
*P. philadelphica* L.	ISSR	Genetic diversity	*Genetic Resources and Crop Evolution*	Zamora-Tavares et al., 2015
Seven *Physalis* species	SNP, InDel	Genetic diversity	*Plant Gene*	Garzon-Martinez, et al., 2015
*P. philadelphica* L.	SNP	Genetic diversity	*Molecular Breeding*	Labate and Robertson, 2015
Eight *Physalis* species	SNP	Genetic diversity	*PLoS One*	Enciso-Rodriguez et al., 2020
*P. philadelphica* L.	SNP	Genetic diversity	*Genetic Resources and Crop Evolution*	Alcala-Gomez et al., 2022
35 *Physalis* species	nrDNA ITS, *Waxy*	Phylogenetics	*Systematic Botany*	Whitson and Manos, 2005
*P. peruviana* L./*P. angulata* L.	ITS, ITS2	Phylogenetics	*SABRAO Journal of Breeding and Genetics*	Jalab and Al-Rufaye, 2024
64 *Physalis accessions*	ITS2, *rbcL*	Molecular authentication and phylogenetics	*Crops*	Pere et al., 2023
33 *Physalis* species	*matK*, *rbcL*, *ndhF*, *rpl32-trnL*, and *ycf1*, ITS and *Waxy*	Phylogenetics	*Molecular Phylogenetics and Evolution*	Zamora-Tavares et al., 2016

Some DNA-based markers, such as simple sequence repeat (SSR), random amplified microsatellites (RAMS), start codon targeted (SCoT), and sequence-characterized amplified region (SCAR), have also shown excellent performance in plant molecular identification. Simbaqueba et al. (2011) identified 1,520 SSRs in the assembled leaf transcriptome of *Physalis peruviana* and developed 138 SSR primer pairs that successfully amplified in *P. peruviana* L. and *Physalis floridana* Rydb. genotypes, with a polymorphism rate of 22%. Delgado-Alvarado et al. (2018) applied RAMS markers to authenticate the varieties and landraces of *Physalis ixocarpa* Brot. ex Hornem. The results indicated that RAMS could be used as good specific markers not only to distinguish *P. ixocarpa* from its close relatives but also to provide specific fingerprints for the authentication of different varieties of *P. ixocarpa*. Feng et al. (2018b) developed four specific SCAR markers for *P. angulata*, *Physalis minima* L., *Physalis pubescens* L., and *Physalis alkekengi* var. *franchetii* (Mast.) Makino based on polymorphism analysis of SCoT molecular markers providing a new method for rapid and accurate molecular identification of the four *Physalis* species.

## Genetic diversity

Research on genetic diversity is crucial for species management planning, as the preservation of diversity plays a vital role in conservation and the breeding of superior individuals. In recent years, there has been a focus on studying the genetic diversity of *Physalis* plants, with several related studies being reported. Various types of DNA molecular markers, including inter-simple sequence repeats (ISSR), random amplified polymorphic DNA (RAPD), SSR, insertion and deletion (InDel), and single nucleotide polymorphism (SNP) markers, have been utilized in numerous studies to assess the genetic diversity and population dynamics of *Physalis* plants. A comprehensive summary of these studies on the genetic diversity of *Physalis* plants can be found in **Table 1**.

RAPD markers, a relatively early molecular marker technology, have been widely utilized in studying genetic diversity in various plants due to their simple operation, high versatility, and cost effectiveness. Hidayat et al. (2017) used RAPD markers to assess the genetic diversity of 23 P*. angulata* plants from different regions of Bandung. Similarly, Khan et al. (2019) employed eight RAPD markers to determine the genetic diversity of 17 accessions of *P. ixocarpa*, with the results aligning with the ecological distribution of accessions and highlighting two accessions (P1512005 and PI360740) from Mexico and Ecuador as exhibiting the highest genetic diversity among *P. ixocarpa* accessions.

Microsatellites, also known as simple sequence repeats (SSRs), are designed based on conserved nucleotide sequences found on both sides of simple repeat sequences, widely distributed in plant genomes (Tautz, 1989). SSRs are co-dominant, multi-allelic, highly polymorphic, and have been widely used in various fields, including genetic diversity, phylogenetic studies, molecular identification, and genetic mapping (Poczai et al., 2013). Several SSR markers have been developed and extensively utilized in the investigation of genetic diversity within *Physalis* species (Simbaqueba et al., 2011; Wei et al., 2012; Delgado-Bastidas et al., 2019; Feng et al., 2023). In a study by Delgado-Bastidas et al. (2019), six SSR markers were employed to evaluate the genetic diversity of 40 genotypes of *P. peruviana* revealing that these genotypes were categorized into three populations. However, it was observed that the level of genetic diversity among the genotypes was notably low, with no discernible population structure. In a more recent study, Feng et al. (2023) developed a set of SSR markers based on chloroplast genome and applied them to assess the genetic diversity and population structure of *P. angulata*. The SSR analysis revealed that 16 populations of *P. angulata* formed four clusters displaying significant geography-related population structure as well as extensive admixture.

ISSR markers are molecular markers that utilize microsatellite oligonucleotides as primers, with two to four randomly selected nucleotides added to the 5′ or 3′ end of the SSR to facilitate annealing at specific sites. The result in PCR amplification of DNA fragments located between relatively spaced repeats that are complementary to the anchor primers (Zietkiewicz et al., 1994). The ISSR, which integrates the advantages of RAPD and SSR, not only exhibits excellent stability and polymorphism but also offers simplicity, rapidity, and efficiency. It has been successfully employed in assessing genetic diversity, genetic relationship, and molecular identification in plants (Wang et al., 2009; Kumar et al., 2018; Tyagi et al., 2020). The ISSR marker has been proven to be valuable in the analysis of genetic diversity and genetic relationships within *Physalis* plants (Vargas-Ponce et al., 2011; Zamora-Tavares et al., 2015). Vargas-Ponce et al. (2011) showed that 12 samples from eight *Physalis* species could be grouped into two clusters with an interspecific genetic similarity ranging from 0.48 to 0.58 based on ISSR analysis. Meanwhile, Zamora-Tavares et al. (2015) utilized 88 ISSR markers to study the genetic diversity and structure of nine *Physalis philadelphica* Lam. populations in western Mexico, revealing high genetic diversity among the samples and grouping the populations into two clusters based on structure analysis.

Single nucleotide polymorphism (SNP) is a widely utilized DNA marker technology that has been developed in recent years. It represents a common genetic variation caused by the alteration of a single nucleotide (A, T, C, and G) in the DNA sequence (Uppu et al., 2018). SNP markers are prevalent in genomes and hold significant value for applications such as plant genetic diversity analysis, genotype identification, high-density genetic map construction, and molecular marker-assisted breeding (Lu et al., 2018; Arca et al., 2020; Guo et al., 2021; Park et al., 2022). Additionally, SNP is one of the most popular molecular marker techniques used to study genetic diversity in *Physalis* plants, as demonstrated by several studies (Cely et al., 2015; Labate and Robertson, 2015; Enciso-Rodriguez et al., 2020; Alcala-Gomez et al., 2022). For example, Enciso-Rodriguez et al. (2020) identified 7,425 SNPs based on Genotyping-By-Sequencing (GBS) and utilized them to assess the diversity of *P. peruviana* and related taxa. Their findings revealed significant gene flow (F_ST_: 0.01–0.05) in different subpopulations of *P. peruviana*. Similarly, Alcala-Gomez et al. (2022) investigated the genetic diversity of *P. philadelphica* using 270 SNP markers based on their study of 40 samples.

## Molecular phylogenetics

The taxonomy of *Physalis* is considered to be a highly complex issue within the Solanaceae due to the significant intraspecific morphological variation and substantial interspecific similarity (Axelius, 1996; Sullivan, 2004; Whitson and Manos, 2005; Olmstead et al., 2008; Pretz and Deanna, 2020). In recent years, molecular analyses have yielded new insights into this problem (Whitson and Manos, 2005; Olmstead et al., 2008; Feng et al., 2016; Zamora-Tavares et al., 2016; Feng et al., 2020; Pere et al., 2023). ITS regions of nrDNA are widely used for studying phylogenic relationships among angiosperms, including the genus *Physalis*, at the interspecific and infrageneric level. This is due to their biparental inheritance, simplicity, universality, intra-genome consistency, inter-genome variability, and high copy number (Whitson and Manos, 2005; Xiang et al., 2013; Feng et al., 2016; Zamora-Tavares et al., 2016; Pere et al., 2023; Jalab and Al-Rufaye, 2024). Whitson and Manos (2005) conducted a study on the phylogenetic relationships among 35 species of *Physalis* and the relationships among the genera of the subtribe Physalinae utilizing the sequence analysis of the nrDNA ITS region and the nuclear gene *waxy*. The findings revealed that the morphologically typical *Physalis* species formed a strongly supported clade. However, the morphologically atypical species, such as *P. alkekengi* L., *Physalis carpenter* Riddell, and *Physalis microphysa* A.Gray were found to be distantly related to any other *Physalis* species resulting in paraphyly within the genus. Zamora-Tavares et al. (2016) utilized five plastids (*matK*, *rbcL*, *ndhF*, *rpl32-trnL*, and *ycf1*) and two nuclear regions (ITS and *waxy*) to examine the phylogenetic relationships of 50 species within the Physalinae, which included 33 *Physalis* species. The study assessed the phylogenetic relationships among recognized genera in Physalinae, with a focus on identifying monophyletic groups and resolving the physaloid grade. Additionally, the study analyzed potential causes for recent divergence within Physalinae. All the aforementioned studies utilized single or a few gene sequence fragments from the plastid genome or nuclear genome to investigate the phylogeny of genus *Physalis*. Due to the limited length of these DNA sequences and their restricted genetic information, there are significant limitations in studying phylogenetic evolution using these methods. We are confident that the ongoing advancements in chloroplast genome and mitochondrial genome-sequencing technology will lead to a more refined and precise reconstruction of the phylogenetic tree of genus *Physalis*.

## Identification of functional genes

### Functional genes involved in inflated calyx syndrome development

The inflated calyx syndrome, also known as the Chinese lantern, is a post-floral morphological novelty in *Physalis* plants (Hu and Saedler, 2007). During the fruit ripening process, the green calyx expands, inflates, and completely envelops the fruit (de Souza et al., 2022). Despite extensive research on this morphological feature of *Physalis* plants, only a limited number of functional genes involved in its development have been investigated and cloned from *Physalis* plants (Wilf et al., 2017). The ortholog of *Solanum tuberosum MADS16* in *P. pubescens*, *MPF2* is a floral tissue-specific expressed gene that is essential for the development of inflated calyx syndrome (He and Saedler, 2005). Furthermore, an MPF2-binding protein, MAGO NASHI, was identified using the yeast two-hybrid system. Two MAGO-encoding genes, *PFMAGPO1* and *PFMAGPO2*, were discovered in *P. floridana*. These genes play a role in male fertility and the evolution of calyx development in *Physalis* (He et al., 2007). Promoter analysis of an *MPF2-like* gene revealed degenerative mutations in its core CArG-box indicating an interaction between floral development and hormone pathways during the development process of calyx inflation syndrome (Khan et al., 2012). MPF3, a core eudicot APETALA1-like MADS-domain protein, has been reported to act as a repressor of *MPF2* during the development of floral calyx identity and inflated calyx syndrome in *Physalis* (Zhao et al., 2013).

Recently, CRISPR-Cas9-targeted mutagenesis technology was utilized for a forward genetics screen to identify the purported essential regulators of inflated calyx syndrome. For instance, the mutation of an *AP2-like* gene has been found to result in a lack of inflated calyx syndrome (He et al., 2023). This technological breakthrough positions *Physalis* as a new model crop for studying fruit development and ripening (Lopez-Gomollon, 2023).

### Functional genes involved in organ growth

*Physalis* fruits are increasingly gaining popularity due to their outstanding sensory and functional characteristics as a functional food (Avendaño et al., 2022). It is important to note that *Physalis* fruit serves as a significant supplementary source of bioactive compounds with high antioxidant activity (Vaillant et al., 2021). Fruit size is a critical quality characteristic of *Physalis* fruit, and there is significant variation in berry sizes among *Physalis* plants. Consequently, the *Physalis* genus is utilized as a model plant for identifying the regulators that may contribute to their variation in berry size (Wang et al., 2012). In *P. philadelphica*, the expression level of *Physalis Organ Size 1* (*POS1*) gene is positively associated with variations in fruit size (Wang et al., 2014). *POS1* plays a crucial role in regulating fruit size by controlling cell wall expansion in Physaleae (Wang et al., 2022). In *P. floridana*, the *Cell Number Regulator 1* (CNR1) gene encodes a cell membrane-anchored modulator that negatively regulates fruit size through its interaction with an AGAMOUS-like ovary identity protein (PfAG2) (Li and He, 2015). Two C-class MADS-domain AGAMOUS-like genes, *PfAG1* and *PfAG2*, in *P. floridana* play essential roles in regulating fruit size and the development process of Chinese lantern (Zhao et al., 2021). Recently, CRISPR-Cas9 technology has been used to target the CLV1 gene revealing its essential role in enhancing fruit size by increasing the number of locules in *P. pruinosa* L (Lemmon et al., 2018).

In addition to regulating the inflated calyx syndrome and fruit development, *P. floridana MPF1* also influences plant architecture, seed development, and flowering time by regulating the expression of *PFLFY*, *PFSOC1*, and *PFFT* genes (He et al., 2010). The *CRABS CLAW* gene in *P. floridana* alters carpel meristem determinacy and carpel closure by mediating the neofunctionalization of *GLOBOSA* genes belonging to the floral B-function MADS-box family (Gong et al., 2021). Additionally, four core exon junction complex core genes in *P. floridana*—namely, *PFMAGO*, *PFY14*, *PFeIF4AIII*, and *PFBTZ*—have been found to play diverse developmental roles in carpel functionality and environmental stress responses. Furthermore, an intron retention in the transcript of *DYT1* was detected in the mutated flowers of *P. floridana* indicating its significance in floral development (Gong et al., 2018). These works provide us with candidate genes for studying the growth and development of *Physalis* plants.

### Functional genes involved in active ingredient metabolism

**Figure 1:**
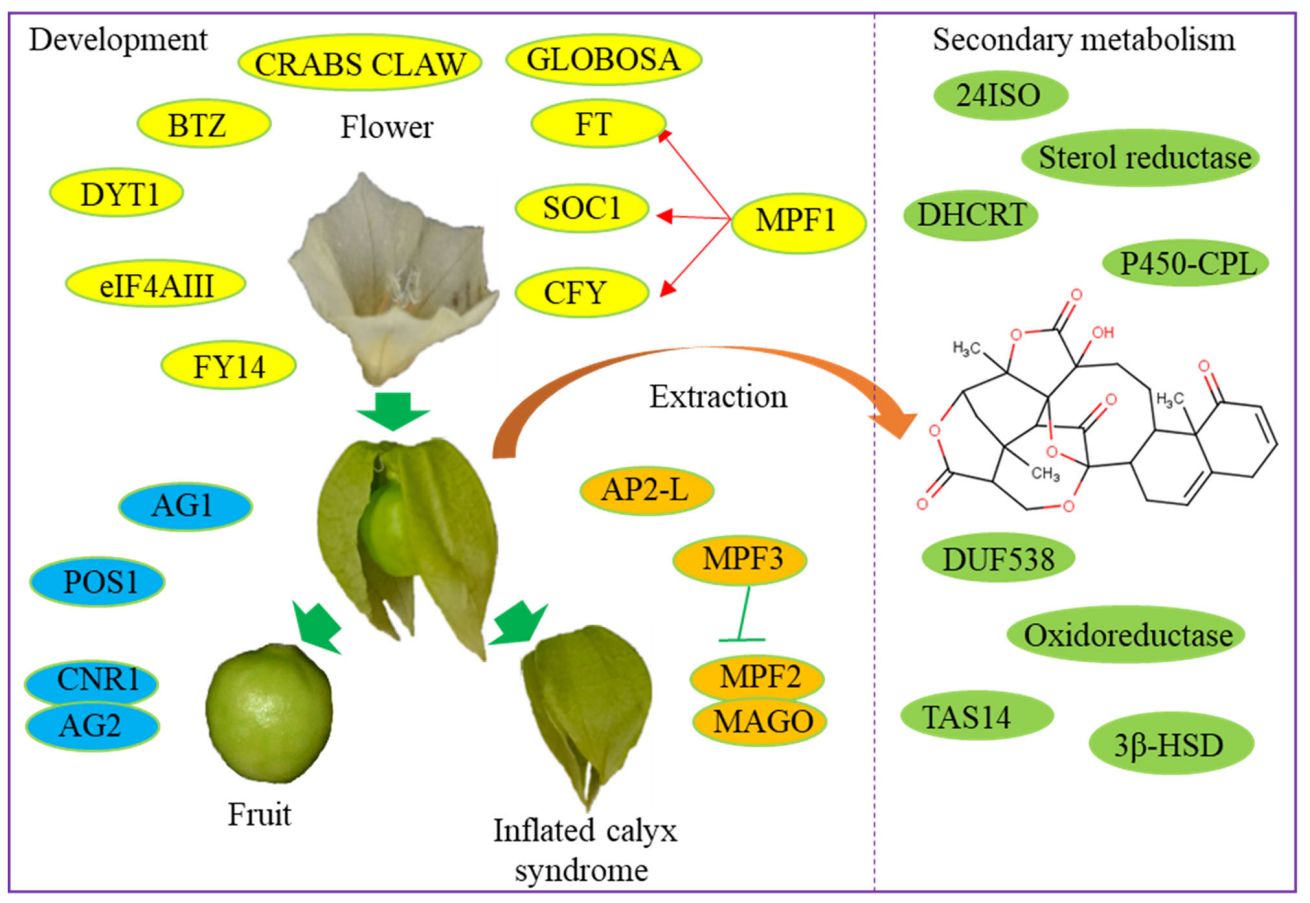
Functional genes involved in the development and secondary metabolism of *Physalis* varieties.

*Physalis* plants produce edible fruits containing numerous antioxidants and bioactive metabolites, including steroidal lactones, withanolides, and physalins (Popova et al., 2022). However, the biosynthesis pathways for these bioactive compounds remain largely unclear. Utilizing *Physalis* transcriptomes, several research groups have identified a variety of genes associated with terpenoid backbone and steroid biosynthesis pathways. In *P. alkekengi*, candidate genes for the oxidation at the C-15/18 positions of steroid backbone required in physalin biosynthesis include a *CYP450* chloroplastic-like gene (unigene-ID: c13295_g2_i2) and an oxidoreductase-like gene (unigene-ID: c16207_g5_i1). Additionally, a gene encoding sterol reductase (c27112_g1_i1) has been identified to be involved in the biosynthesis of specialized metabolites in *P. peruviana* (Fukushima et al., 2016). *Pa24ISO* catalyzes the isomerization of 24-methylenecholesterol to 24-methyldesmosterol in the physalin biosynthesis process (Yang et al., 2022). *DHCR7* from *P. angulata* was newly identified through heterologous expression. Heterologous expression of *P. angulata DHCR7* in *Saccharomyces cerevisiae* confirmed its role in producing 24-methylene-cholesterol, a key substrate in the physalin and withanolide biosynthesis pathway (Yang et al., 2021). In *P. angulata*, DUF538 was predicted as a positive regulator and TAS14 as a negative regulator in the regulation of physalin biosynthesis (Zhan et al., 2020). These functional genes have potential implications for accelerating the breeding of high-yielding *Physalis* varieties rich in bioactive compounds (**Figure 1**).

## Application of omics in *Physalis* study

Several omic datasets of *Physalis* plants have been made available online offering extensive genetic information for the screening of functional genes and the identification of active compounds. In the present review, all the omic datasets of *Physalis* plants are summarized in **Tables 2**, **3**.

**Table 2:** The detailed information of all the transcriptomes, metabolomes. and proteomes of *Physalis* plants.

Species	Tissue	Omics type	Publication	Group/year
*P. peruviana* L.	Leaf	Transcriptome	*BMC Genomics*	Garzón-Martínez et al., 2012
*P. floridana* Rydb.	Flower/fruit	Transcriptome	*Planta*	Gao et al., 2020
*P. philadelphica* L.	Reproductive organ	Transcriptome	*J Exp Bot.*	Wang et al., 2012
*P. alkekengi* L.*/P. peruviana* L.	Leaf	Transcriptome	*Front Plant Sci.*	Fukushima et al., 2016
*P. angulata* L.	Hairy root	Transcriptome	*Plant Mol Biol.*	Zhan et al., 2020
*P. peruviana* L.	Root/stem	Transcriptome	*Peer J.*	Garzón-Martínez et al., 2021
*P. angulata* L.	Root/stem/leaf/flower/fruit	Transcriptome	*Plant Signal Behav.*	Lu et al., 2019
*P. peruviana* L.	Fruit	Metabolome	*Food Chem.*	Llano et al., 2018
*P. peruviana* L.	Fruit	Metabolome	*Food Chem.*	Maruenda et al., 2018
*P. angulata* L.	Hairy root	Metabolome and Proteome	*J. Agric. Food Chem.*	Zhan et al., 2018
*P. peruviana* L.	Fruit	Metabolome	*J Food Sci.*	Yu et al., 2019
*P. pruinosa* L.	Fruit/calyx/leaf/stem/root	Metabolome	*Food Res Int.*	Mahana et al., 2022
*P. peruviana* L.	Seedling	Metabolome	*Molecules*	Monroy-Velandia and Coy-Barrera, 2021
*P. angulata* L./*P. grisea* (Waterf.) M. Martínez/*P. philadelphica* L.	Leaf	Metabolome	*Plant Signal Behav.*	Trujillo-Pahua et al., 2021
*P. angulata* L.	Fruit	Metabolome	*Molecules*	Lima et al., 2020
*P. alkekengi* L.	Calyx/fruit	Metabolome	*Antioxidants*	Crescenzi et al., 2023
*P. peruviana* L.	Seedling	Metabolome	*Molecules*	Monroy-Velandia and Coy-Barrera, 2021
*P. philadelphica* Lam.	Leaf	Metabolome	*Pest Manag Sci.*	Meza-Canales et al., 2022
*P. alkekengi* L.	fruit	Proteome	*Se Pu*	Yu et al., 2013

**Table 3:** The summaries of complete chloroplast genomes of *Physalis* species.

*Physalis* species	GenBank accession	Genome size (bp)
*P. chenopodiifolia* Lam.	MN508249.	156,900
*P. philadelphica* Lam.	MT254545	156,804
	MN192191	156,804
	MZ539568	156,856
*P. minima* L.	MH045577	156,692
*P. pubescens* L.	MH045576	157,007
*P. cordata* Houst. ex Mill.	ON018728	157,000
*P. angulata* L.	MH045574	156,905
	MH019241	156,706
*P. angulata* var. *villosa* Bonati	OM257167	156,898
*P. pruinosa* L.	MH019243	156,706
*P. peruviana* L.	MH019242	156,706
	OP028208	156,715
	KP295964	156,706
*P. alkekengi* var. *franchetii* (Mast.) Makino	MH045575	156,578
*P. macrophysa* Lam.	OP748222	156,735
*P. ixocarpa* Brot. ex Hornem	OP748223	156,871

### Transcriptomic analysis

In recent years, advancements in the next-generation sequencing technology have enabled the identification of functional genes in non-model plants even without genomic data (Su et al., 2011). The first *Physalis* transcriptome was published in 2012 using fresh leaf tissue from the Colombian ecotype of Cape gooseberry (*P. peruviana*), as plant material, generating a number of assembled sequences and candidate markers (Garzón-Martínez et al., 2012). To study the *Physalis*–*Fusarium oxysporum* pathosystem, the transcriptome of *P. peruviana* was further utilized to identify genes related to immunity, including 74 resistance genes, 17 receptor-like kinase genes, 8 PAMP-triggered immunity genes, and 9 effector-triggered immunity genes (Enciso-Rodriguez et al., 2013). Comparative transcriptomic analysis of *P. philadelphica* with different sizes of reproductive organs resulted in the identification of 263 differentially expressed transcripts (Wang et al., 2012). Using the RNA-seq method, 75,221 genes of *P. alkekengi* and 54,513 genes of *P. peruviana* were identified. The authors discovered numerous genes potentially involved in each step of the terpenoid backbone and steroid biosynthesis pathway providing new insights into the intricate chemical and structural diversity of *Physalis* plants (Fukushima et al., 2016). In *P. angulata*, a well-known traditional Chinese medicine with various active compounds, transcriptomic approaches were used to screen genes involved in the biosynthesis of bioactive compounds (Zhan et al., 2020). A transcriptomic analysis revealed 468 unigenes involved in the flower–fruit transition process in *P. floridana* uncovering some potential genetic variations that contribute to the early stage of fruit development in *Physalis* (Gao et al., 2020). These studies in *P. angulata* can help spur our understanding of the biosynthetic pathways underlying key metabolites important to medicine and plant development.

### Metabolomic analysis

Untargeted metabolomics is a recently developed method that offers a streamlined approach to systematically analyze and compare the differences in primary and secondary metabolites among different groups (Souard et al., 2018). Using untargeted metabolomics, Medina’s group identified several specifically accumulated withanolides and fatty acyl glycosides as molecular markers to differentiate between organic and conventional *P. peruviana* fruits (Llano et al., 2018). NMR-based metabolomic analysis revealed significant phytochemical variations in *P. peruviana* fruits (Maruenda et al., 2018). In *P. angulata* hairy roots, a comparative metabolomic analysis revealed variations in the contents of physalins D and H under MeJA treatment suggesting a possible regulatory mechanism underlying the MeJA-induced biosynthesis of active compounds (Zhan et al., 2018, 2020). LC-MS/MS-based metabolomic analysis revealed variations in carotenoid content during different growth stages of *P. peruviana* fruit (Yu et al., 2019). Metabolite profiling using UPLC-MS identified a total of 293 metabolites, including 61 terpenoids, 58 phenolic acids, and 53 flavonoids, in aqueous and ethanolic extracts of Amazonian fruits (including *P. angulata*) (Lima et al., 2020). Metabolomics, in combination with chemometrics, has identified several potential α-glucosidase and α-amylase inhibitory metabolites in *P. pruinosa*. Physangulide B, physaperuvin G, and neophysalin A were found to be positively correlated with α-glucosidase inhibition activity, while guaiacyl-primeveroside, phyperunolide C, and perulactone were found to be positively correlated with α-amylase inhibitory activity (Mahana et al., 2022). Using LC-ESI/LTQOrbitrap/MS followed by LC-ESI/LTQOrbitrap/MS/MS technique, 58 phytocompounds were identified in the calyx ad fruit of yellow *P. alkekengi* (Crescenzi et al., 2023). Metabolomic analysis has been utilized to investigate the responses of *Physalis* species to environmental stimuli. In *P. peruviana*, the upregulation of a free flavonol during different growth stages indicates a response to salt stress (Monroy-Velandia and Coy-Barrera, 2021). Metabolomic analysis of three different *Physalis* species revealed several species-specific metabolites following larval herbivory. In *P. angulata*, the response to herbivory is highlighted by the upregulating of various compounds, such as withanolide, α-trehalose, and cimiracemoside D. Pheophorbide A and azamacrocycle are common metabolites of *P. grisea* (Waterf.) M. Martínez and *P. philadelphica* that are responsive to herbivory (Trujillo-Pahua et al., 2021). Husk tomato (*P. philadelphica*) seedlings are susceptible to infestation by the whitefly *Trialeurodes vaporariorum*. A newly published metabolome study showed that *P. philadelphica* impairs whitefly development by inducing significant changes in metabolic profiles (Meza-Canales et al., 2022). Metabolomics researches provide us an opportunity to understand the differences in types and contents of active ingredients in *Physalis* plants. Metabolomic analysis is also an effective way to screen novel varieties with high medicinal ingredients.

### Proteomic analysis

MS/MS-based peptide sequencing techniques have been utilized for the large-scale identification and screening of differentially produced proteins (Yates et al., 1993). In 2013, protein extracted from *P. alkekengi* fruit was analyzed by nano-RPLC-MS/MS system with shotgun proteomics method providing the foundation for further investigation into the functional proteins in *Physali*s species (Yu et al., 2013). MeJA is commonly employed as a chemical elicitor to enhance the accumulation levels of various bioactive metabolites in plants (Liu et al., 2016). Proteomic analysis revealed that several terpenoid and steroid biosynthesis-related enzymes, such as CYP monooxygenases and 3β-hydroxysterioid dehydrogenase, might be the targets of the MeJA-induced active ingredient biosynthesis (Zhan et al., 2018). Enzyme engineering is currently a hot topic in biotechnology. Proteomic analysis helps us to identify key enzymes involved in the biosynthesis of active ingredients and improve their activities by enzyme engineering modifications.

### Complete chloroplast genomic analysis

The chloroplast plays crucial roles in various cellular functions, such as photosynthesis, signal transduction, and stress response (Martin Avila et al., 2016). The examination of the complete chloroplast genomes of the *Physalis* genus will be useful for in-deep genetic research. Currently, the complete chloroplast genomes of 12 *Physalis* species, including *Physalis chenopodiifolia* Lam., *P. angulata*, *P. angulata* var. *villosa* Bonati, *P. alkekengi*, *P. minima*, *P. pubescens*, *P. peruviana*, *P. pruinosa*, *Physalis cordata* Houst. ex Mill., *P. philadelphica*, *Physalis macrophysa* Rydb., and *P. ixocarpa* were available (Sandoval-Padilla et al., 2019; Zamora-Tavares et al., 2019; Feng et al., 2020; Zhan et al., 2022; Sandoval-Padilla et al., 2022a, b; Zhang et al., 2023) (**Table 3**). The complete chloroplast genomes mentioned above ranged in size from 156,578 to 157,007 bp, with the number of protein coding genes ranging from 79 to 80 and the number of tRNA genes ranging from 30 to 31 (Sandoval-Padilla et al., 2019; Zamora-Tavares et al., 2019; Feng et al., 2020; Sandoval-Padilla et al., 2022a, b). These publicly available chloroplast genomes enable effective phylogeography and phylogenetic studies of *Physalis*. Furthermore, a significant number of SSR loci have been identified providing precise molecular markers for investigating the intraspecific diversity of *Physalis*.

### Chromosome-level genomic analysis

Genome-editing technologies have been developed to enhance the quality and yield of crops, improve adaptation to diverse environments, manipulate plant architecture and fruit size, and broaden the range of staple crops that can be cultivated (Scheben et al., 2017). Although most of the *Physalis* species have a similar chromosome number and structure to Solanaceae, genomic knowledge is essential for genome editing in *Physalis* crops. The genome of the orphan crop *P. pruinosa* was first sequenced and published in 2018 producing 66.3 Gb of raw data (Lemmon et al., 2018). To gain a deeper better understanding of the genetic variations that contribute to the origin and diversity of these distinctive traits, high-quality genomes of classic *Physalis* genus plants were published (Lu et al., 2021). *P. floridana* possesses an assembled genome size of 1,389 Mb, which serves as a valuable resource for breeding *Physalis* crops (Lu et al., 2021). Recently, a chromosome-scale references for *P. grisea* and its close relative *P. pruinosa* were published providing high-quality genome assemblies for genome editing in *Physalis* species (He et al., 2023). To fully understand the genomic variation of *Physalis* plants, a high-quality haplotype-resolved genome will greatly promote the understanding of the complex traits of *Physalis* plants.

## Future perspectives

Researchers have performed many studies on the screening of germplasm collections and identifying SNPs with continuously updating genetic techniques. Although several molecular markers have been developed, outdated platforms provide a limited ability to estimate the extent of *Physalis* genetic variability. In the future, a number of convenient and phenotype-based molecular markers will definitely be the direction for efficient genetic diversity analysis and germplasm resource identification. Genetic identification is a prerequisite for species conservation and resource utilization. Furthermore, the advancement of high-throughput sequencing technology will lead to an increasing number of published chloroplast genomes and mitochondrial genomes, thus contributing to the further development of phylogenetic tree reconstruction for genus *Physalis*.

Single-cell RNA sequencing (scRNA-seq) is a novel technology used to investigate cell heterogeneity at a high resolution (Bawa et al., 2022). Mass spectrometry (MS) imaging is a recently developed MS-based metabolomics approach to reveal the distribution of metabolites at the spatial level (Xiang et al., 2022; Zhan et al., 2024). However, scRNA-seq and MS imaging have not yet been applied to the study of *Physalis* plants. ScRNA-seq will provide novel insights into the inflated calyx syndrome and fruit development of *Physalis* plants. High-resolution MS imaging can be utilized to identify and visualize metabolic heterogeneity, including inflated calyx syndrome. Future researches on *Physalis* plants will move toward higher resolutions at both the temporal and spatial levels.

The recently sequenced genome of *P. floridana* acts as a starting point for genome-enabled research (Lu et al., 2021). As more species’ genomes are fully sequenced, new knowledge regarding evolutionary relationships, processes, and patterns, as well as the ability to map biosynthetic pathways through comparative means will emerge.

## Author contributions

YJ: Writing – original draft, Data curation, Investigation. YYJ: Writing – original draft, Investigation, Methodology. YS: Writing – original draft, Investigation, Methodology. QZ: Writing – original draft, Investigation, Data curation. HW: Writing – original draft, Funding acquisition, Resources. CS: Methodology, Writing – review & editing. SF: Writing – original draft, Writing – review & editing, Project administration, Funding acquisition.
